# Individual Biometric Identification Using Multi-Cycle Electrocardiographic Waveform Patterns

**DOI:** 10.3390/s18041005

**Published:** 2018-03-28

**Authors:** Wonki Lee, Seulgee Kim, Daeeun Kim

**Affiliations:** School of Electrical and Electronic Engineering, Yonsei University, 50 Yonsei-ro, Seodaemun-gu, Seoul 120-749, Korea; wonkilee@yonsei.ac.kr (W.L.); slgee@yonsei.ac.kr (S.K.)

**Keywords:** electrocardiography, individual biometric identification, multi-cycle ECG waveform, pattern matching

## Abstract

The electrocardiogram (ECG) waveform conveys information regarding the electrical property of the heart. The patterns vary depending on the individual heart characteristics. ECG features can be potentially used for biometric recognition. This study presents a new method using the entire ECG waveform pattern for matching and demonstrates that the approach can potentially be employed for individual biometric identification. Multi-cycle ECG signals were assessed using an ECG measuring circuit, and three electrodes can be patched on the wrists or fingers for considering various measurements. For biometric identification, our-fold cross validation was used in the experiments for assessing how the results of a statistical analysis will generalize to an independent data set. Four different pattern matching algorithms, i.e., cosine similarity, cross correlation, city block distance, and Euclidean distances, were tested to compare the individual identification performances with a single channel of ECG signal (3-wire ECG). To evaluate the pattern matching for biometric identification, the ECG recordings for each subject were partitioned into training and test set. The suggested method obtained a maximum performance of 89.9% accuracy with two heartbeats of ECG signals measured on the wrist and 93.3% accuracy with three heartbeats for 55 subjects. The performance rate with ECG signals measured on the fingers improved up to 99.3% with two heartbeats and 100% with three heartbeats of signals for 20 subjects.

## 1. Introduction

Individual identification is an important issue for security. However, the traditional methods using identification cards or certificates have concerns regarding their loss or unauthorized copy. As an alternative, biometric approaches have recently received much attention for individual identification. The typical biometric technologies currently available are fingerprint identification [1,2,3], face identification [4,5], and iris identification [6]. Among various biometric information generated by the human body, ECG signals, which monitor the electrical heart activity, are another possible solution for individual identification. ECG signals can potentially identify each individual uniquely depending on the position, shape, size, and structure of the heart. The features observed in ECG signals remain unchanged by human will. In addition, they can be measured easily without reluctance, and signal identification is robust from contamination, wear, and forgery. Because of these characteristics, the use of ECG signals for biometric recognition studies has recently received much attention, and extracting ECG features has been challenging for biometric recognition [7].

ECG signals can be measured by recording the electrical activity of the heart over a period using electrodes placed on a subject’s skin. They convey a large amount of information on the structure of the heart and the function of its electrical conduction system [8]. ECG signals can also be used to assess the rate and rhythm of heartbeats, size and position of the heart chambers, presence of any damage to the heart’s muscle cells or conduction system, effects of cardiac drugs, and function of implanted pacemakers [9]. For this tendency, ECG signals are recorded for the assessment of cardiac function and have been used to detect abnormal patterns of heart activity.

Typically, electrodes are directly attached to the skin. However, recently, non-contact electrode methods for ECG data acquisition have been considered. For clinical use, the use of dry and noncontact electrodes received investment [10] and the method of noncontact monitoring of cardiorespiratory activity by electromagnetic coupling with human tissue was considered [11]. Currently, a contactless capacitive sensory system for the detection of ECG-like signals [12] is considered. The acquisition approach is based on a capacitive coupling with the patient body performed by electrodes integrated in a front-end circuit and the proposed system is able to detect changes in the electric charge related to the heart activity. These electrodes may be a more comfortable tool for biometric identification. Furthermore, they show satisfactory results in particular applications, such as in the automotive environment [13] or in wearable health devices [14]. ECG signals sensed from mobile devices can be potentially applied for biometric identification with remote access control [15,16].

As a biometric tool, ECG signals were analyzed as an automatic ECG classification system based on the principal component analysis (PCA) to interpret 12 uncorrelated clinical diagnosis features related to the P, QRS, and T amplitudes and durations [17]. For identification, there have been approaches of detecting fiducial points and using the inter-relations or characteristics of feature points, for example, intervals between the feature points, such as R-R interval and ST-T duration. Israel et al. [18] used 15 temporal features related to the P-QRS-T segments for individual identification. Wang et al. [19] merged temporal signals and amplitudes related to the fiducial points for analysis. Nor et al. [20] used the amplitude values of the Q, R, and S waves as features and demonstrated that the use of three fiducial points is sufficient to identify a subject, in contrast to the common practice of using more feature points. Extracting the features is crucial for classification, and the performance depends on the accurate localization of wave boundaries with the P-QRS-T segment. For that reason, there have been many attempts to eliminate noise using frequency-selective filters or wavelet de-noising. However, owing to the characteristics of the ECG signals in the presence of noise, detecting the P wave is still challenging; nevertheless, the QRS and T wave detection techniques have started to provide acceptable results in most cases [21,22]. In addition, heartbeats change, and the signals may fluctuate or be influenced by physical activities, drug consumption, and strong emotions; in this case, the identification process would become more difficult than that under static body conditions.

To overcome this limitation, fiducial-independent approaches that analyze the overall morphology have been proposed. Agrafioti and Hatzinakos [23] proposed a method for individual identification by applying autocorrelation of 5-s ECG segments. Wübbeler et al. [24] showed a method based on the distance between the first and second temporal derivatives of signals. Poree et al. [25] used the maximal correlation coefficient applied over a 12-lead ECG, and a cross correlation of a 12-lead ECG was used for human verification [26] and identification [27]. Many researchers have suggested various sophisticated algorithms to classify the ECG signals, e.g., neural networks [20,28,29], independent component analysis [30], k-nearest neighborhood [31,32,33,34], and support vector machine [35,36,37,38,39]. Recently, multi-modal identifications based on data fusion and dimensionality reduction have been investigated. Bugdol and Mitas [40] proposed a combination of sounds and ECG signals as behavioral biometric features. Dimensionality reduction methods using the PCA method have been used to optimize the feature set, and the nearest neighbor classifier yields the highest identification accuracy. Wang et al. [41] presented a feature reduction method combining PCA with linear discriminant analysis and a probabilistic neural network classifier to discriminate eight different types of arrhythmia from ECG beats. He and Tan [42] developed a new entropy-based PCA approach for dimensionality reduction. Zapata et al. [43] summarized the state-of-the-art data fusion oriented to biometric authentication and identification, exploring its techniques, benefits, advantages, disadvantages, and challenges. An interesting work is to observe the amplitude values of the Q, R, and S waves to identify an individual, not the intervals between fiducial points [20]. It is consistent with our idea that one signal cycle is re-sampled and mapped into a regular interval of segments by ignoring the temporal period information.

Many conventional methods have focused on feature extraction using the QRS complex, P wave, and T wave in a cycle of the ECG signal. Detecting all the feature points precisely and identifying each segment are deeply related to individual identification. In this study, we propose a holistic approach to apply ECG signal pattern matching without using all the feature points. We introduce a new method using matching of a standard frame of multi-cycle ECG waveforms for individual biometric identification. We also develop an easy measurement of ECG signals with electrodes patched on the fingers as well as on the wrist. The details are described in the following sections.

## 2. Method

**Figure 1:**
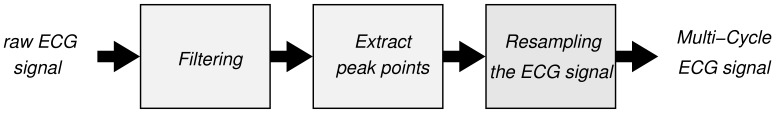
Multi-stage process for acquiring a standard frame of multi-cycle ECG signal.

For the individual identification using the ECG signals, a multi-stage process was required to change the raw ECG signal to a standard frame of multi-cycle ECG signal. The overall diagram for obtaining the multi-cycle ECG signal is shown in Figure 1. Herein, we introduce how the raw ECG signals were transformed into a regular form for pattern matching as well as how several matching algorithms work for individual identification.

### 2.1. ECG Signal Acquisition

**Figure 2:**
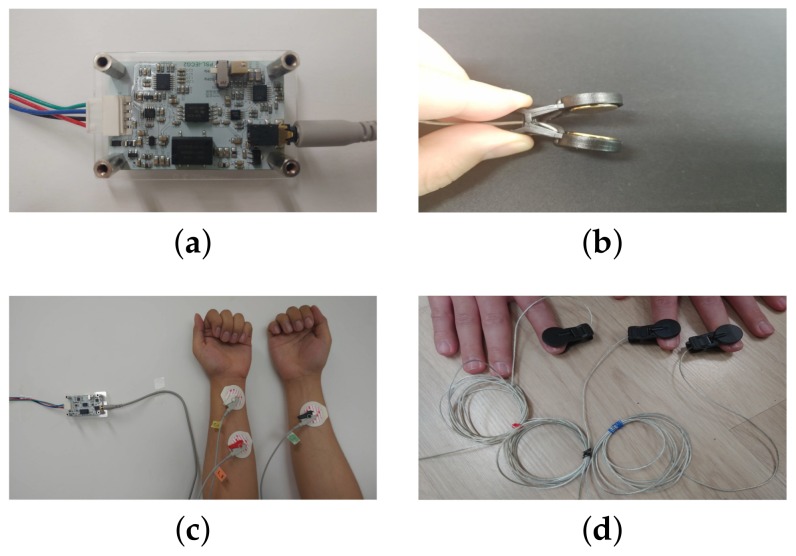
ECG signal measuring circuit and two type of electrodes on the wrist and on the fingers: (**a**) ECG measuring circuit; (**b**) electrode as a button style (which can be patched on the fingers); (**c**) measuring ECG signals on the wrist; (**d**) measuring ECG signals on the fingers.

The ECG signals were acquired using an ECG measuring circuit, a data acquisition (DAQ) board, and a personal computer. The ECG measuring circuit is a one-channel ECG analog amplifier module applying DC 5 V to the power input. For ECG signal acquisition, three electrodes were patched on either the wrists or fingers. First, we recorded the ECG signals using three disposable electrodes attached to the skin of the wrists (3-wire ECG). The developed circuit and electrodes (two on the left and one on the right) are shown in Figure 2. The voltage between the (positive) left arm electrode and right arm electrode was measured. For considering an easier measurement of the ECG signal and its authentication, we designed another ECG interface for the fingers. Figure 2d shows the snapshots of the measurement of the ECG signals from three fingers, using the electrode shown in Figure 2b. The ECG signals from three separate fingers were less noisy than the ECG signals with two electrodes on the same finger.

**Figure 3:**
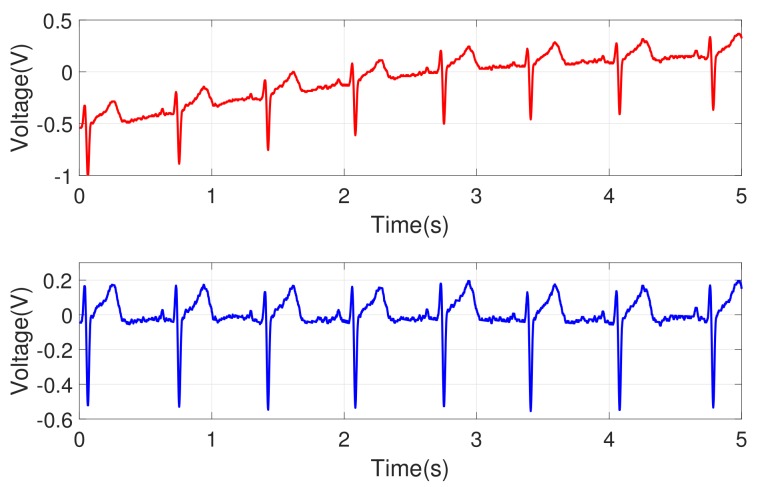
Example of ECG signals after a band-pass filter was applied: (**top**) raw ECG signal; (**bottom**) signal after filtering is applied (note that the increasing transition of baseline is removed).

**Figure 4:**
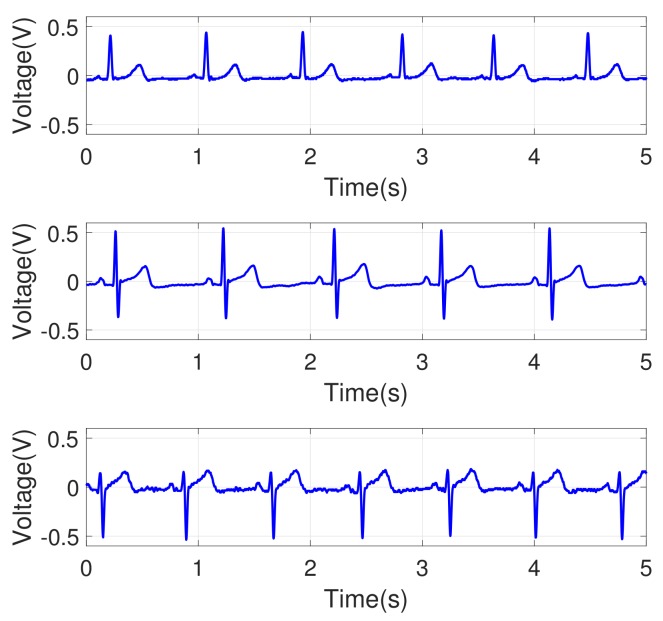
Examples of ECG signals after band-pass filtering. Each subfigure shows the results of three different individuals.

The noise removal process was needed to extract the refined ECG signals. Power supply noise and high-frequency noise were observed in an original ECG signal. Those noises were removed via a band-pass filter with a frequency range of 0.3 Hz to 35 Hz, and they were implemented in analog. The circuit’s internal amplification was designed with a 750-fold amplifier (±2%) to have an output range of 0∼3.3 V and the amplified signal was sampled with a rate of 500 Hz. The measured signals from the circuit can still show overall patterns that are not intrinsic to the data. For example, a slow transition of baseline signal was observed as in Figure 3 (top). To eliminate that, a low-order polynomial (order 6) was fitted to and subtracted from the signal. Figure 3 (bottom) shows the ECG signal after filtering, and Figure 4 shows the signals for three different individuals. We observed that each individual had his/her own ECG waveform pattern.

#### 2.1.1. Extracting Peak Points

To classify the standard frame of the ECG signals, positive high peak points were selected as delimiters, which are the most remarkable points detected without errors even in noisy signals. A cycle of the ECG signal can be obtained from peak to peak. In our experiments, a cycle of the ECG signal was sampled between two positive R-R peak points. To find the positive peak points, differentiating the noise-removed signal and then squaring the differentiated signal are needed. Then, after applying the moving average filter of order 5, we could find the peak points of the ECG signal.

**Figure 5:**
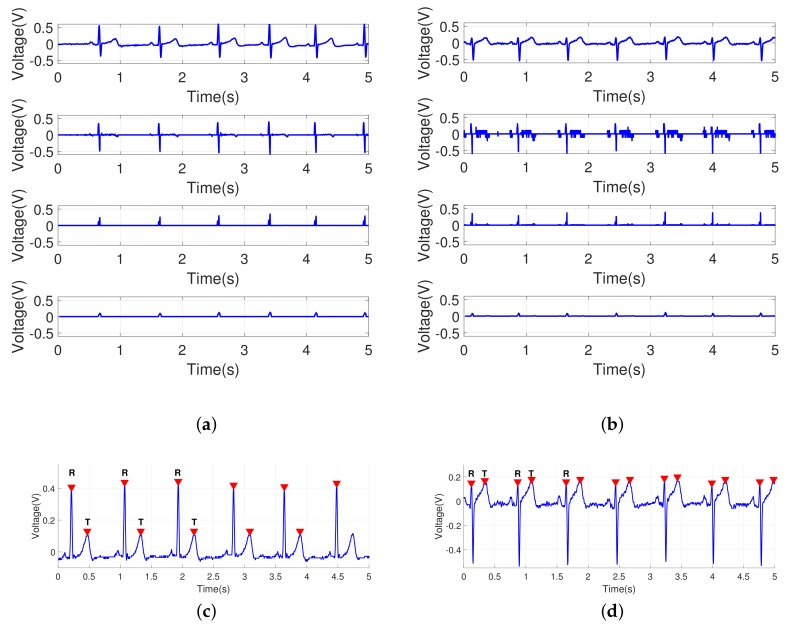
Extracting the peak points for two different ECG signals: (**a**,**b**) results of the sequential process for extracting the peak points; signal after band-pass filtering, differentiated signal, squared signal, and signal passing through the moving average filter; (**c**) detected peak points (R and T points) for the ECG signal in Figure 4 (top); (**d**) detected peak points (R and T points) for the ECG signal in Figure 4 (bottom); another pass of scanning is needed to determine the T points after a pair of R points are found.

Between a pair of peak points (R’s), the second positive highest peak can be found without difficulty by calculating the differentiation over a given cycle of signal, which is mapped to the midst of 100 samples. In the experiments, the second highest peak as another dominant feature was searched within one cycle, which corresponds to the T wave in the ECG signal. Thus, the R-T and T-R intervals can be monitored in the re-sampled signals. Here, the P wave is not our main concern in the experiments because it may not be a salient feature for some individuals. The overall process of detecting the peak points is shown in Figure 5.

#### 2.1.2. Re-Sampling Process

For the ECG signal pattern matching, we used a re-sampling process for the ECG signals to frame an arbitrary length of signal into a regular interval of the same length. One cycle of the ECG waveform cut out the QRS complex at the position of the R wave, since the peak point at R was selected as a delimiter in a cycle. It may lose information around the QRS complex interval. To include the shape of the QRS complex interval for identification, more than one cycle of signals is required. We can select at least two cycles (two heartbeats) of the peak-to-peak ECG signals and then we build a ECG vector whose length is 100 for a cycle and 200 for two cycles.

**Figure 6:**
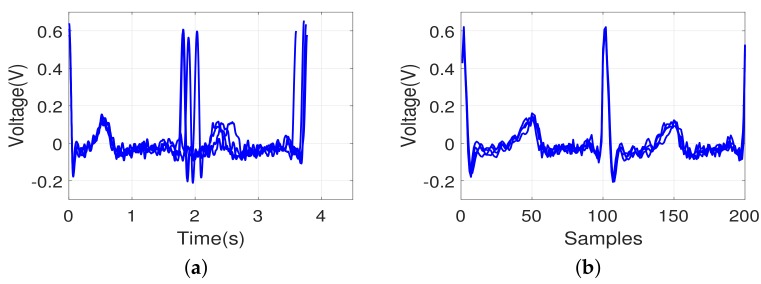
Example of an ECG pattern, including two heartbeats, shown by an overlapping of four different consecutive heartbeats: (**a**) original signal between the R-R-R fiducial points; (**b**) signal after re-sampling to a 200-point pattern.

Between two R points (starting and ending points) in a cycle, a peak (T wave) can be observed. In the re-sampling process, the following segments can be ideally built based on the points: two segments R∼T, T∼R for a cycle, and four segments R∼T, T∼R, R∼T, T∼R for two cycles. For a two-cycle signal, each of four segments is mapped into the same size of interval: 1∼50, 51∼100, 101∼150, and 151∼200. That is, each segment is re-sampled uniformly into the interval of 50 samples. Even the same individual’s signal may change within consecutive heartbeats. The temporal positions of the local peak points, such as the T wave observed in the middle of a cycle, are not exactly equal in all the signals. The above re-sampling process will produce a standard frame of the ECG vector. Figure 6 shows an example of an ECG pattern, including two heartbeats, shown by an overlapping of four different consecutive heartbeats. We noted that the period of the same individual’s original ECG signals varied, and the above re-sampling process by interpolation yielded a uniform ECG waveform pattern.

### 2.2. Pattern Matching Algorithms

For identification, the ECG signals were classified using four-fold cross validation. A collection of ECG patterns were randomly partitioned into four bins of subsamples. Of the four equal-sized subsamples, a bin of subsamples was retained as the validation data for testing, and the other three bins were used as the training data. The cross validation process was then repeated for four folds. The above test procedure was repeated four times with a random order of ECG patterns. The performance results were averaged to estimate the identification rate.

Every ECG signal is mapped into the same length of samples, which forms an ECG vector, and a pair of ECG vectors can be compared using a similarity measure. We tested four different pattern matching algorithms, i.e., cosine similarity, cross correlation, city block distance, and Euclidean distance. They are explained in the following subsections in order.

#### 2.2.1. Cosine Similarity

Cosine similarity measures the similarity between two vectors as an inner product space that calculates the cosine of the angle between them. It can be represented using a dot product and magnitude of the two vectors as follows:

(1) $$d_{cosine}\left(A,B\right)=\frac{A\cdot B}{∥A∥\;∥B∥}=\frac{\sum_{i=1}^{N}A_{i}\times B_{i}}{\sqrt{\sum_{i=1}^{N}{\left(A_{i}\right)}^{2}}\sqrt{\sum_{i=1}^{N}{\left(B_{i}\right)}^{2}}}$$

where *N* is the number of samples, *A* is the training data, and *B* is the test data. The signal with the highest score value is selected as the most similar with an input signal.

The similarity range is bounded in [0 1], which indicates that it is exactly the opposite of the reference vector, to 1, which indicates that it is exactly the same as the vector, with 0 indicating orthogonality (de-correlation) and in-between values indicating intermediate similarity or dissimilarity. The metric is a judgment of orientation and not magnitude.

#### 2.2.2. Cross Correlation

Cross correlation is a measure of similarity of two series signals as a function of the lag of one relative to the other. It is also known as a sliding dot product or sliding inner-product and commonly used for searching a long signal using shorter and known features. Cross correlations are useful for determining the time delay between two signals, while sample cross correlations are used to determine the degree of similarity between the input signal and the database signal. For data pairs, $\left(A^{\prime },B^{\prime }\right)=\left\{\left(A_{1},B_{1}\right),\left(A_{2},B_{2}\right),\ldots ,\left(A_{N},B_{N}\right)\right\}$, an estimate of the cross covariance with the delay *k* was defined as follows:

(2) $$d_{cross}\left(A,B\right)\left(k\right)=\frac{f_{AB}\left(k\right)}{\sqrt{f_{AA}\left(0\right)}\sqrt{f_{BB}\left(0\right)}},\;k=0,\pm 1,\pm 2,\ldots ,\pm T$$

where $\sqrt{f_{AA}\left(0\right)}$ and $\sqrt{f_{BB}\left(0\right)}$ are variations of *A* and *B*, respectively, and

(3) $$f_{AB}\left(k\right)=\left\{\begin{array}{c} \begin{array}{c} \frac{1}{N}\sum_{i=1}^{N-k}\left(A_{i}-\bar{A}\right)\left(B_{i+k}-\bar{B}\right),\;k=0,1,2,\ldots ,N\\ \frac{1}{N}\sum_{i=1}^{N+k}\left(B_{i}-\bar{B}\right)\left(A_{i-k}-\bar{A}\right),\;k=0,-1,-2,\ldots ,-N \end{array} \end{array}\right.$$

with $\bar{A}$ and $\bar{B}$ as the sample means of the series.

#### 2.2.3. City Block Distance

The city block distance can be calculated as follows:

(4) $$d_{city\_block}\left(A,B\right)=\sum_{i=1}^{N}\left|A_{i}-B_{i}\right|$$

The calculated value would be zero if two signals are identical and greater than zero if there is a minimal similarity. It shows a similar result with the Euclidean distance in most cases. However, the degree of difference is not squared, and the effect of a large difference is reduced.

#### 2.2.4. Euclidean Distance

The Euclidean distance is the length of the line segment connecting two points [44]. In the Euclidean space, it can be employed to find the most similar signal with the input using the following equation:

(5) $$\begin{array}{c} d_{Euclidean}\left(A,B\right)=\sqrt{\sum_{i=1}^{N}{\left(A_{i}-B_{i}\right)}^{2}} \end{array}$$

### 2.3. ECG Database

For the experiments, the ECG signals of 55 healthy individuals aged 19–26 years were measured. Each of the 55 individuals were assessed four times under the same physical conditions on the same date. The recording duration was 10 s per individual recording, and the time interval between recordings was 10 min. However, invalid cycles within the recorded signals were observed for some subjects and more than three consecutive cycles were rarely available for those subjects. Each extracted ECG signal was re-sampled as shown in Figure 6b for individual comparison. In the matching experiment, the database comprised 220 re-sampled ECG waveform patterns (each individual had four ECG signals). One signal among the four signals from the same individual was selected as a test set, and the other ECG waveforms were averaged for comparison. The selected signal was considered as the input, and the averaged template of the remaining signals was considered as the training set. The summarized statistics of the data used to test the proposed algorithm are shown in Table 1a.

To determine whether the finger ECG data could be used as the input signals to the algorithm, a new ECG database was obtained from 20 healthy individuals, which was collected on the same date, but was different from the wrist ECG database tested above as shown in Table 1b.

## 3. Results

### 3.1. Identification with Multi-Cycle ECG Data

**Figure 7:**
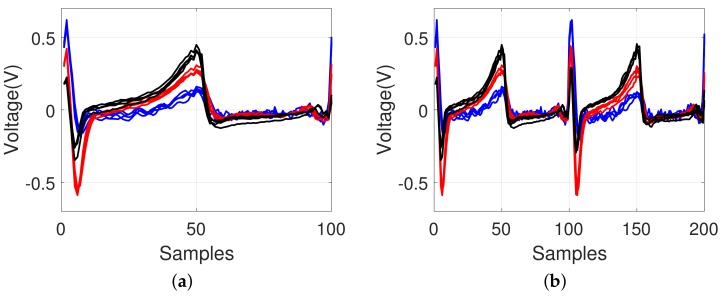
Example of ECG pattern from three different individuals (signals from the same individual are collected from the same recording session and they are closely overlapped): (**a**) one heartbeat; (**b**) two heartbeats. Different color lines show about different individuals.

We used four different measures for pattern matching as described above. The aim of the pattern matching in this study was human identification, i.e., identification of a subject in the test dataset by searching the minimal distance with a pattern in the training dataset. To determine the effect of the pattern matching measures using the multi-cycle signals, the performance using one signal cycle was also tested. Figure 7 shows overlapping ECG signals for three different individuals. The same individual’s ECG waveform patterns were consistent as expected. The waveform patterns varied among the subjects. This tendency implies that the ECG pattern matching method could be used for individual identification.

The performances with the four matching algorithms using the wrist ECG data were evaluated; the results are shown in Table 2. The identification rate *I* as the evaluation measure is calculated as follows:

(6) $$I=\sum_{i}x_{i}/N$$

where $x_{i}$ is 1 or 0 depending on whether the *i*-th ECG test sample is correctly identified for its subject, and *N* is the total number of test samples.

Based on the results, we confirmed that our proposed method can possibly be applied for individual identification. With the Euclidean distance measure, the identification rate was 93.28%. This method showed the highest identification rate. With the cosine similarity measure, the identification rate was 85.85%; it had the worst performance among the tested methods. The accuracy increased to 92.69% with the city block measure. The method using the three-cycle signals showed a better performance than that using the one- or two-cycle signals regardless of the matching algorithms. The multi-cycle signals conveyed better information for the ECG classification.

Table 3 shows the identification rates with the different pattern matching methods using the finger ECG data. The identification rate increased to 99.34% with two cycles (heartbeats) and 100% with three cycles (heartbeats). The performance cannot be directly compared with that of the individual identification experiment on the 55 individuals as shown in Table 2a. The number of subjects was different, and both results could not be directly compared. Table 2b shows the results for the reduced number of 20 subjects, which can be compared to the evaluation of the finger-based ECG identification with the same number of subjects. The identification accuracy in the 20 subjects was better than that in the 55 subjects because of the different population size. It seems that the smaller the population is, the greater the identification accuracy is. Furthermore, the finger ECG data with the suggested method showed a higher performance.

### 3.2. Identification with Various Types of Signal Segments

In the proposed algorithm, we detected the R peak over the ECG signal, extracted one cycle of the signals, and mapped this segment into 100 samples. Similarly, two or three consecutive cycles of ECG signals can be mapped into exactly 200 or 300 samples as a regular frame interval. However, the T, R, or P waves can be candidate boundary points for a cycle of a signal. For one cycle, the R-R interval or the T-T interval can be used; the P peak point may not be easily detectable for some ECG signals. As such, we did not assess the P-P interval. In addition, the R-T segment or the T-R segment within one cycle can be selected for identification. Table 4 shows the classification performances depending on the different types of segments. With the T wave or R wave selected as the starting and ending points to form a cycle, the T-T interval and the R-R interval had a similar performance because a set of segments was commonly included. It seems that using only a partial segment information of the ECG signal has a limitation. The performance rate decreased to 66.71% with the R-T segment and 82.28% with the T-R segment. We can infer that the T-P-Q-R segment in Figure 1 provided more information for identification than the R-S-T segment.

## 4. Discussion

### 4.1. Comparison with Other Methods

In this paper, we suggest a method using the entire pattern of the ECG waveforms. The peak-to-peak signals were re-sampled into a regular interval for a cycle. Thus, this process removed the signal distortion due to fast or slow heartbeats. With the selection of peak points, even multi-cycle ECG signals can be mapped into a uniform frame. We considered an easy measurement of ECG signals by patching electrodes on the fingers. According to the results, a high biometric identification rate can be achieved without a feature analysis.

In this study, we showed that more cycles of ECG signals can better train a variation of signals for biometric identification. Even if one signal cycle is deviated from the standard form, another signal may fit into the standard form. The multi-cycle signals had higher probability to be recognized as the same subject. In that case, the identification performance with multi-cycles can be improved. The results of the authentication or individual identification using the ECG signals are summarized in Table 5. The proposed approach and other state-of-the-art methods (different databases) were compared, and our approach showed a reasonably good performance compared to other methods. From that argument, identifying the proper number of cycles would be an interesting topic. Owing to the limited length of the obtained valid ECG signals, much longer cycles (more than three cycles) were not tested in this study.

### 4.2. Effect of Different Body Conditions

**Figure 8:**
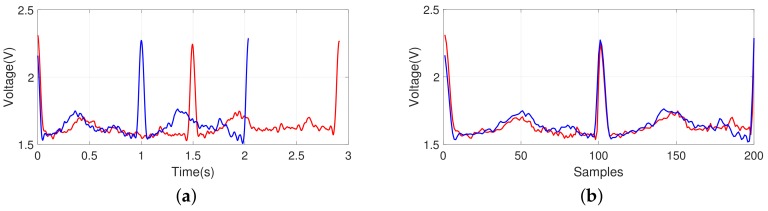
Example of an ECG pattern shown by an overlapping to compare the changes under different physical conditions on different dates for the same individual; (**a**) before re-sampling and (**b**) after re-sampling (red-colored line: after, blue-colored line: before).

Figure 8 shows how the identification is affected using ECG waveforms for the same individual under different physical conditions on different dates. As shown in Figure 8a, the ECG cycle became shorter after 10 min of physical exercises, running, and holding of breath for a certain period on different dates. However, owing to the re-sampling process in our model, the impact of the heartbeat changes was minimized because different durations of the two-cycle signals were normalized as shown in Figure 8b, which was classified into the same individual.

**Figure 9:**
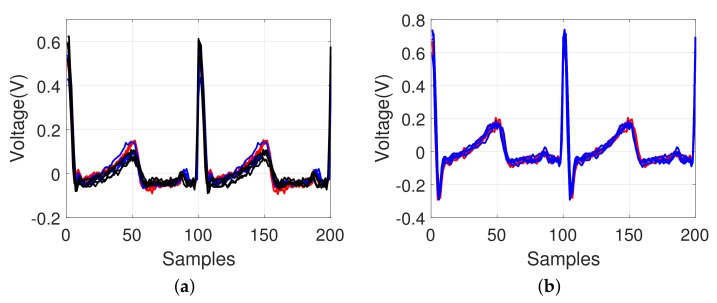
Examples of ECG pattern shown by an overlapping to compare the changes after some period or after smoking; (**a**) after some period (red-colored line: reference, blue-colored line: after 7 days, black-colored line: after 20 days) and (**b**) before and after smoking (red-colored line: after, blue-colored line: before).

In addition, as shown in Figure 9a, it was confirmed that there was a minimal change in the ECG waveform patterns in a given frame interval even after many days (after 7 days and 20 days). For our re-sampled model, the ECG waveform patterns remain unchanged; this indicates that it is possible to use the method for individual identification. When we investigated the ECG patterns of smokers, there was also a minimal change in the ECG signals before and after smoking as shown in Figure 9b.

### 4.3. Effect of Possible Heart Disorders

**Figure 10:**
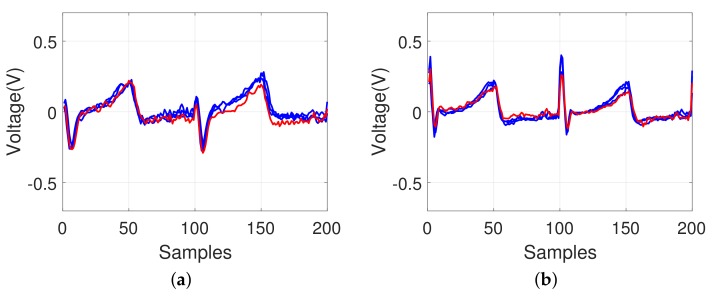
Examples of ECG pattern shown by an overlapping of the unrecognized signals (red-colored line: recognized as a different identity from the same subject). Each subfigure shows the different ECG signals that failed to be recognized.

Figure 10 shows examples of the ECG signals that failed to be recognized (red-colored line). Most of the signals showed only minimal variations, which can be interpreted to indicate the same individual. However, some signals showed comparable differences that can be distinguished from the majority of the signals for the same individual data, which was failed for identification. This may be because of the body movement of the subjects, abnormal condition of the heart, or similar disturbances. These abnormal signals might provide information regarding the symptoms by correlation to the body condition (possible heart disorders), although this is not within the scope of this study.

### 4.4. Alternative Approach

The ECG signal, in addition to the fingerprint and iris, can be an alternative measure for individual identification. Biometric recognition may be exposed to forgery and falsification. The ECG authentication as well as iris or fingerprint identification may have such problems. If there are multiple biometric identification tools, the system can not only enhance the security, but also increase the identification rate. Alternatively, active sensing can be considered, emitting current flow to the skin and sensing the electrical signal reflected from the skin. Estimating or modeling the signals expected from the emitted current flow and comparing them with the sensed signals may prevent the forgery of the ECG signals using an electrical device. Further studies on active sensing for ECG signals are needed.

## 5. Conclusions

We have shown the effect of a longer length of cycles; this indicates that a holistic view of the signal pattern without extracting features can be potentially used for individual identification. In this study, the individual identification reached an identification rate of 93.28% with three cycles of the wrist ECG signals and 99.34% and 100% with two and three cycles of the finger ECG signals, respectively. The suggested method has a reasonable performance compared with the current popular methods, despite its simple matching algorithms. The current equipment used to measure the ECG signal is obviously rudimentary to obtain signals stably and repetitively; however, the proposed method that matches a standard frame of the ECG signals helps in the observation of the characteristics of the individual heart signals being used as an alternative measure for individual identification. A more refined design will be useful for portable individual biometric identification systems, and other techniques, such as wavelets of neural networks and data fusion, could improve the results. Combining the distance metrics of different methods could improve the identification rate. In the future, we will continue to perform experiments using more sophisticated algorithms and consider combining various approaches to enhance the identification rate.

In the ubiquitous network environment, the monitoring system, such as U-health, would be able to check the health of an individual at any moment and at any place. Using the characteristics of biometric signals of an individual, the system can continuously monitor the health and also identify the individual among databases in real time. The above ECG experiments might provide a possible suggestion to the health monitoring system.

## Figures and Tables

**Table 1:** Different recording data: (a) from the wrists; (b) from the fingers.

Information	Data
(a)	
Number of individuals	55 (19∼27 years old)
ECG acquisition method	one channel 3-wire ECG
Number of ECG records	220 records
Number of ECG records for each individual	4 records
Training set	165 records (4-fold cross validation)
Test set	55 records (4-fold cross validation)
(b)	
Number of individuals	20 (19∼23 years old)
ECG acquisition method	one channel 3-wire ECG
Number of ECG records	80 records
Number of ECG records for each individual	4 records
Training set	80 records (4-fold cross validation)
Test set	20 records (4-fold cross validation)

**Table 2:** Identification rates with the four different pattern matching methods using wrist ECG data: (a) for 55 subjects; (b) for the reduced number of 20 subjects.

Length of Signal	Cosine	Cross	City Block	Euclidean
(a)				
One cycle	78.18%	83.00%	85.91%	88.75 %
Two cycles	84.89%	85.00%	88.75%	89.89%
Three cycles	85.85%	85.73%	92.69%	93.28%
(b)				
One cycle	87.50%	91.56%	93.75%	95.63%
Two cycles	91.56%	93.44%	96.88%	95.62%
Three cycles	94.69%	96.38%	97.19%	97.50%

**Table 3:** Identification rates with the four different pattern matching methods using finger ECG data for 20 subjects.

Length of Signal	Cosine	Cross	City Block	Euclidean
One cycle	93.42%	93.09%	98.68%	98.36%
Two cycles	96.38%	97.36%	98.68%	99.34%
Three cycles	96.38%	96.38%	98.68%	100.00%

**Table 4:** Identification rates with different segments using wrist ECG data for 55 subjects.

Feature	R-R Interval	T-T Interval	R-R Interval	T-T Interval	R-T Segment	T-R Segment
Num. of cycles	2 cycles	2 cycles	1 cycle	1 cycle	1 cycle	1 cycle
Euclidean	89.89%	89.78%	88.75%	87.97%	66.71%	82.28%

**Table 5:** Comparison between the proposed method and other approaches.

Method	Database	Accuracy
Israel et al. (2005) [18]	self-db: 29 subjects	100%
Wübbeler et al. (2007) [24]	self-db: 74 subjects	98.1%
Wang et al. (2008) [19]	MIT-BIH normal sinus	100%
Agrafioti and Hatzinakos (2009)	MIT-BIH normal sinus	96.2%
Lourenco et al. (2011) [45]	self-db: 16 subjects	94.3%
Poree et al. (2011) [25]	self-db: 11 subjects	91.4%
Zokaee and Faez (2012) [33]	MIT-BIH db	96.2%
Lee et al. (2012) [46]	self-db: 10 subjects	99.5%
Zhao et al. (2013) [34]	PTB db: 12 subjects	96.0%
Jekova and Bortolan (2015) [47]	Test PTB db: 14 subjects	77.6%
Jekova et al. (2018) [27]	self-db: 20 subjects	97.2%
Jekova et al. (2018) [27]	self-db: 50 subjects	94.5%
Our method (two cycles of signals from the wrist)	self-db: 55 subjects	89.9%
Our method (three cycles of signals from the wrist)	self-db: 55 subjects	93.3%
Our method (two cycles of signals from the wrist)	self-db: 20 subjects	95.6%
Our method (three cycles of signals from the wrist)	self-db: 20 subjects	97.5%
Our method (two cycles of signals from the fingers)	self-db: 20 subjects	99.3%
Our method (three cycles of signals from the fingers)	self-db: 20 subjects	100%
